# Association between changes in HBsAb levels and hepatic adverse events after HBsAg clearance in non-cirrhotic chronic hepatitis B patients

**DOI:** 10.1186/s12879-026-13791-9

**Published:** 2026-06-26

**Authors:** Wen Deng, Xiaoxue Chen, Ziyu Zhang, Xinxin Li, Weihua Cao, Yaqin Zhang, Shiyu Wang, Linmei Yao, Xin Wei, Shuojie Wang, Zixuan Gao, Yao Xie, Minghui Li

**Affiliations:** 1 Department of Hepatology Division 2, Beijing Ditan Hospital, Capital Medical University, 8 Jing Shun East Street, Chaoyang District, Beijing, 100015 China; 2 HBV Infection, Clinical Cure and Immunology Joint Laboratory, Capital Medical University, Beijing, 100069 China; 3 Department of Hepatology Division 2, Peking University Ditan Teaching Hospital, Beijing, 100015 China

**Keywords:** Chronic hepatitis B, Hepatitis B surface antigen (HBsAg), Hepatitis B Surface antibody (HBsAb), Pegylated interferon-alpha (Peg-IFNα)

## Abstract

**Objective:**

The relationship between changes in hepatitis B surface antibody (HBsAb) levels and hepatic adverse events after hepatitis B surface antigen (HBsAg) clearance remains unclear. This study aimed to investigate the correlation between HBsAb level changes and hepatic adverse events in non-cirrhotic patients following HBsAg clearance.

**Methods:**

We retrospectively analyzed patients with HBsAg seroclearance achieved via pegylated interferon-alpha (Peg-IFNα) therapy in the Department of Hepatology II at Beijing Ditan Hospital, Capital Medical University, from October 2008 to December 2023. Participants were stratified by baseline HBsAb levels into negative (< 10 mIU/mL), low (10–100 mIU/mL), medium (100–1000 mIU/mL), and high (≥ 1000 mIU/mL) groups. Based on HBsAb trends during follow-up, patients were categorized into declining, stable, and rising groups. The primary endpoint was the incidence of hepatic adverse events after HBsAg clearance.

**Results:**

A total of 390 patients were included, with a median age of 38 (32–44) years and a median follow-up of 53.50 (11.00–173.00) months. During follow-up, 4 cases of hepatic adverse events occurred, all in the declining group (4 cases, 3.05%), while none were observed in the stable or rising groups (*P* = 0.018). Changes in HBsAb levels were inversely correlated with the incidence of hepatic adverse events (*rs* = -0.125, *P* = 0.014).

**Conclusion:**

Declining HBsAb levels after HBsAg clearance may be associated with an increased risk of hepatic adverse events.

**Clinical trial:**

This study was registered at ClinicalTrials.gov (ID: NCT04301908; registration date: 03/06/2020; https://register.clinicaltrials.gov/).

## Introduction

Chronic hepatitis B virus (HBV) infection remains a major global public health concern and is a leading cause of liver cirrhosis, hepatocellular carcinoma (HCC), and hepatic decompensation [1, 2]. To reduce the risk of adverse hepatic outcomes in individuals with HBV infection, current guidelines recommend achieving a functional cure—defined as hepatitis B surface antigen (HBsAg) seroclearance, with or without hepatitis B surface antibody (HBsAb) seroconversion, undetectable hepatitis B virus (HBV) DNA, hepatitis B e antigen (HBeAg) loss, and sustained virological and biochemical remission for at least 24 weeks after treatment cessation—as the optimal clinical treatment endpoint [1, 3, 4]. Although HBsAg seroclearance significantly reduces the incidence of liver-related complications such as cirrhosis and HCC in clinical practice, some patients may still develop these outcomes [5, 6]. To date, the factors influencing adverse liver events after HBsAg seroclearance remain incompletely understood.

HBsAb, a protective antibody, provides long-term immune protection by mediating the clearance of circulating HBsAg and viral particles, and it may contribute to the durability of HBsAg seroclearance after treatment withdrawal [7]. However, the correlation between HBsAb levels and the occurrence of adverse liver events following HBsAg seroclearance remains unclear.

This study aims to explore the relationship between HBsAb levels, their dynamic changes during post-treatment follow-up, and the incidence of adverse hepatic events in patients who achieved HBsAg seroclearance through interferon-based therapy. The findings may provide predictive indicators for long-term outcomes following functional cure.

## Materials and methods

### Research subjects

Patients who achieved HBsAg seroclearance following pegylated interferon-alpha (Peg-IFNα) therapy at the Liver Disease Center of Beijing Ditan Hospital, Capital Medical University, between 2008 and 2023 were included. We had access to information that could identify individual participants during data collection. Inclusion Criteria: (1) HBsAg < 0.05 IU/mL, HBV DNA < 20 IU/mL, and HBeAg negativity after Peg-IFNα treatment; (2) Post-treatment follow-up duration ≥ 48 weeks after HBsAg seroclearance and discontinuation of therapy; (3) Age 18–65 years. Exclusion Criteria: (1) Coexisting liver diseases, including hepatitis A, C, D, or E, Alcoholic liver disease (ALD), autoimmune hepatitis, or non-alcoholic fatty liver disease (NAFLD); (2) Concurrent infections with other viruses causing liver injury, such as Epstein-Barr virus (EBV), cytomegalovirus (CMV), or human immunodeficiency virus (HIV); (3) Presence of cirrhosis at the time of HBsAg seroclearance (defined as a Fibrosis Index Based on 4 factors (FIB-4) score ≥ 3.25); (4) Diagnosis of malignancies at enrollment.

This study was conducted in accordance with the principles of the Declaration of Helsinki. The study protocol was approved by the Ethics Committee of Beijing Ditan Hospital, Capital Medical University (Approval No.: Jing Di Lun Yan Zi [2023] No. (009)-01) and registered at ClinicalTrials.gov (ID: NCT04301908; registration date: 03/06/2020; https://register.clinicaltrials.gov/).

This was a retrospective study, and the ethics committee waived the requirement for informed consent.

### Data collection

Data were collected through the Hospital Information System (HIS) and Laboratory Information System (LIS), including patients’ demographic data (age, gender), treatment information (antiviral treatment regimens, discontinuation time), baseline data at Peg-IFNα treatment cessation (defined as data collected when patients had both achieved HBsAg seroclearance and discontinued treatment), including HBsAb levels, HBV DNA levels, liver function tests, imaging results, and other relevant parameters, and endpoint follow-up data, including the timing and type of liver-related adverse events, the occurrence of newly developed liver cirrhosis, and the date of the last follow-up. Baseline was defined as the time of Peg-IFNα treatment discontinuation following HBsAg seroclearance, and the endpoint as the time of the first liver-related adverse event or the last follow-up. Liver function parameters, HBV virological and serological indicators were assessed every 3–6 months during follow-up, and hepatic imaging results evaluated every 6–12 months.

Patients in this study were stratified according to baseline HBsAb levels into four groups: negative (< 10 mIU/mL), low (≥ 10 and < 100 mIU/mL), medium (≥ 100 and < 1000 mIU/mL), and high (≥ 1000 mIU/mL).

Additionally, based on changes in HBsAb levels from HBsAg seroclearance (treatment discontinuation) to the end of follow-up, patients were divided into three groups: Decline group (downgrade in HBsAb level category), Stable group (no change in HBsAb level category), Rise group (upgrade in HBsAb level category).

### Laboratory testing

All clinical biochemical and virological indicators were measured at the Clinical Laboratory Center of Beijing Ditan Hospital. HBsAg and HBsAb levels were determined using the Abbott microparticle chemiluminescence immunoassay (Abbott Laboratories, USA; Abbott i2000 automated immunoassay analyzer). The upper detection limits were HBsAg > 250 IU/mL and HBsAb > 1000 mIU/mL, with HBsAb ≥ 10 mIU/mL defined as HBsAb positivity. HBV DNA was quantified using fluorescent quantitative polymerase chain reaction (Roche, Switzerland; Light Cycler 480 PCR system), with a lower detection limit of 20 IU/mL. Liver function tests were performed using the Hitachi 7180 automated biochemical analyzer. HCC and cirrhotic ascites were assessed via color Doppler flow imaging (Siemens, USA; ACUSON X150), computed tomography (Siemens, USA; SOMATOM Definition AS), or magnetic resonance imaging (Siemens, USA; MAGNETOM Skyra).

### Data analysis

Data were analyzed using SPSS 19.0 and visualized with GraphPad Prism. Normally distributed continuous data are presented as mean ± standard deviation and compared using ANOVA or an independent samples t-test. Non-normally distributed continuous data are expressed as median (interquartile range) and analyzed with the Mann-Whitney U test. Paired samples were compared using the paired t-test or the Wilcoxon signed-rank test for non-normal data. Categorical data are reported as frequency (percentage) and analyzed with the chi-square test. Correlation analyses included: Point-biserial correlation for binary categorical vs. non-normal continuous variables; Phi coefficient for two binary categorical variables; Spearman’s rank correlation for categorical vs. ordinal variables, two ordinal variables, or ordinal vs. continuous variables. All statistical tests were two-tailed, with significance defined as α = 0.05.

### Outcome measures

The incidence of liver-related adverse events (cirrhotic ascites, liver failure, upper gastrointestinal bleeding, and HCC) following HBsAg clearance.

## Results

### Demographic data of patients

A total of 645 patients who achieved HBsAg seroclearance after Peg-IFNα therapy were enrolled. Of these, 189 were excluded due to follow-up durations < 48 weeks, 20 due to missing HBsAb results at the study endpoint, and 46 due to baseline cirrhosis. Ultimately, 390 patients were included in the analysis. The median age at the time of HBsAg seroclearance and treatment discontinuation was 38 (interquartile range: 32, 44) years, with a median follow-up duration of 53.50 (range: 11.00–173.00 months).

### Baseline HBsAb level grouping data

Patients were stratified into four groups based on baseline HBsAb levels: negative group (*n* = 70), low-level group (*n* = 112), intermediate-level group (*n* = 165), and high-level group (*n* = 43). The median ages at HBsAg clearance and treatment discontinuation were 42 (37, 47), 38 (33, 44), 37 (32, 42), and 33 (29, 40) years, respectively, with statistically significant differences among the groups (*H* = 28.519, *P* < 0.001). A correlation analysis between baseline HBsAb levels and age at HBsAg seroclearance revealed a weak negative correlation (*rs* = -0.248, *P* < 0.001). The correlation coefficient matrix for all variables is shown in Table 1.

**Table 1:** Correlation matrix of the variables

Variable	Age	Male	ALT abnormal ^b^	AST abnormal ^b^	TBIL abnormal ^b^	ALB abnormal ^b^	HGB abnormal ^b^	PLT abnormal ^b^	ALT abnormal ^e^	AST abnormal ^e^	TBIL abnormal ^e^	ALB abnormal ^e^	HGB abnormal ^e^	PLT abnormal ^e^	Adverse events	Liver cirrhosis ^e^	HBsAb level classification ^b^
Male	0.127*																
ALT abnormal ^b^	-0.120*	-0.059															
AST abnormal ^b^	-0.119*	-0.083	0.381**														
TBIL abnormal ^b^	-0.024	0.075	-0.003	-0.033													
ALB abnormal ^b^	0.092	-0.019	-0.021	0.025	0.041												
HGB abnormal ^b^	-0.076	-0.101*	0.025	-0.015	0.006	0.056											
PLT abnormal ^b^	0.117*	0.266**	0.107	-0.011	0.023	-0.005	0.094										
ALT abnormal ^e^	-0.002	0.173**	0.144**	0.042	0.075	-0.015	-0.056	0.081									
AST abnormal ^e^	0.046	0.077	0.119*	0.148**	0.007	0.065	0.006	0.001	0.307**								
TBIL abnormal ^e^	0.031	0.164**	-0.080	-0.007	0.229**	0.029	0.034	0.093	0.041	-0.022							
ALB abnormal ^e^	-0.065	-0.191**	-0.040	-0.028	-0.038	-0.022	0.098	-0.015	-0.100	-0.031	-0.071						
HGB abnormal ^e^	-0.024	-0.167**	0.111*	-0.055	0.010	0.069	0.263**	0.041	0.025	-0.039	-0.052	0.062					
PLT abnormal ^e^	0.057	0.071	-0.028	-0.020	0.010	0.167*	0.054	0.135*	0.176**	0.033	0.093	-0.030	0.037				
Adverse events	0.045	-0.041	-0.015	0.018	-0.025	-0.015	-0.038	-0.003	-0.011	-0.020	0.020	-0.016	-0.020	0.254**			
Liver cirrhosis ^e^	0.103*	-0.067	-0.042	0.035	-0.022	0.194	0.056	0.080	0.135*	-0.018	0.114	-0.014	0.141	0.456**	0.282*		
HBsAb level classification ^b^	-0.248**	-0.033	0.124*	0.123*	-0.014	-0.036	-0.039	0.032	0.050	0.100*	-0.015	0.106*	-0.058	0.022	0.083	0.048	
Changes in HBsAb stratification	-0.010	-0.015	-0.041	-0.028	-0.005	-0.035	0.021	-0.001	0.026	-0.034	0.059	-0.119*	0.071	-0.020	-0.125*	-0.032	-0.454**

Note: Alanine Aminotransferase (ALT), Aspartate Aminotransferase (AST), Total Bilirubin (TBIL), Albumin (ALB), Hemoglobin (HGB), Platelet count (PLT), and Hepatitis B surface antibody (HBsAb). The superscript letter “b” indicates data at baseline/HBsAg clearance discontinuation, and the superscript letter “e” indicates data at the endpoint/follow-up conclusion. ALT abnormalityᵇ, AST abnormalityᵇ, TBIL abnormalityᵇ, ALB abnormalityᵇ, HGB abnormalityᵇ, and PLT abnormalityᵇ denote data at HBsAg clearance discontinuation. ALT abnormalityᵉ, AST abnormalityᵉ, TBIL abnormalityᵉ, ALB abnormalityᵉ, HGB abnormalityᵉ, and PLT abnormalityᵉ denote data at follow-up conclusion. HBsAb grading refers to categorization based on baseline HBsAb levels, while HBsAb grading change is categorized according to the shift in HBsAb levels from baseline to endpoint. P value: ** denotes significant correlation at the 0.01 level (two-sided); * indicates significant correlation at the 0.05 level (two-sided)

Baseline alanine aminotransferase (ALT) abnormality rates showed no significant differences among the four groups [35 (50.00%) vs. 58 (51.78%) vs. 100 (60.61%) vs. 30 (69.77%), χ² = 6.379, *P* = 0.095], whereas baseline aspartate aminotransferase (AST) abnormality rates differed significantly [9 (12.86%) vs. 17 (15.18%) vs. 31 (18.79%) vs. 14 (32.56%), χ² = 8.020, *P* = 0.046]. Correlation analysis between baseline HBsAb levels and ALT/AST abnormality rates at HBsAg clearance cessation revealed a weak positive association (rs = 0.124, *P* = 0.014; rs = 0.123, *P* = 0.015). Other baseline liver function parameters—total bilirubin (TBIL), hemoglobin (HGB), platelet count (PLT), and albumin (ALB)—showed no significant intergroup differences (*P* > 0.05).

The overall follow-up duration was 53.50 (11.00–173.00) months, with no significant differences among the negative, low-, moderate-, and high-level groups [50.50 (12.00–173.00), 46.50 (12.00–164.00), 59.00 (12.00–160.00), and 60.00 (11.00–118.00) months, respectively; *H* = 3.689, *P* = 0.297]. Endpoint ALT, AST, TBIL, ALB, HGB, and PLT levels also showed no significant intergroup differences (*P* > 0.05).

By the end of follow-up, 4 patients developed hepatic adverse events. The incidence rates were 0.00% (0/70) in the negative group, 0.89% (1/112) in the low-level group, 0.61% (1/165) in the moderate-level group, and 4.65% (2/43) in the high-level group, with no statistical significance (*χ²* = 4.471, *P* = 0.143). Additionally, 3 patients developed cirrhosis, with no significant intergroup differences in incidence [0.00% (0/70) vs. 0.89% (1/112) vs. 0.61% (1/165) vs. 2.33% (2/43); *χ²* = 2.271, *P* = 0.513]. Detailed patient characteristics are shown in Table 2.

**Table 2:** Patient characteristics by baseline HBsAb levels

Variable	All patients (*n* = 390)	Negative group(*n* = 70)	low-level group (*n* = 112)	moderate-level group (*n* = 165)	high-level group (*n* = 43)	χ2 / H	*P*
Male (n, %)	268(68.72%)	48(68.57%)	79(70.54%)	115(69.70%)	26(60.47%)	1.609	0.657
Age (years)	38.00(32,44)	42(37,47)	38(33,44)	37(32,42)	33(29,40)	28.519	<0.001
Adverse events (n, %)	4(1.03%)	0(0%)	1(0.89%)	1(0.61%)	2(4.65%)	4.471	0.143
Baseline FIB-4 score stratification						1.595	0.661
FIB-4 < 1.45	208(53.33%)	35(50.00%)	62(55.36%)	85(51.52%)	26(60.47%)		
1.45 ≤ FIB-4 < 3.25	182(46.67%)	35(50.00%)	50(44.64%)	80(48.48%)	17(39.53%)		
ALT abnormal ^b^ (n, %)	223(57.18%)	35(50.00%)	58(51.78%)	100(60.61%)	30(69.77%)	6.379	0.095
AST abnormal ^b^ (n, %)	71(18.21%)	9(12.86%)	17(15.18%)	31(18.79%)	14(32.56%)	8.020	0.046
TBIL abnormal ^b^ (n, %)	23(5.90%)	5(7.14%)	5(4.46%)	12(7.27%)	1(2.33%)	1.881	0.576
ALB abnormal ^b^ (n, %)	8(2.05%)	2(2.86%)	3(2.68%)	2(1.21%)	1(2.33%)	1.645	0.680
HGB abnormal ^b^ (n, %)	48(12.31%)	9(12.86%)	16(14.29%)	19(11.52%)	4(9.30%)	0.881	0.830
PLT abnormal ^b^ (n, %)	103(26.41%)	16(22.86%)	27(24.11%)	52(31.52%)	8(18.60%)	4.321	0.230
ALT abnormal ^e^ (n, %)	116(29.74%)	20(28.57%)	31(27.68%)	48(29.10%)	17(39.53%)	2.281	0.516
AST abnormal ^e^ (n, %)	15(3.85%)	0(0%)	4(3.57%)	8(4.85%)	3(6.98%)	4.885	0.152
TBIL abnormal ^e^ (n, %)	68(17.44%)	15(21.43%)	17(15.18%)	28(16.97%)	8(18.60%)	1.237	0.756
ALB abnormal ^e^ (n, %)	9(2.31%)	0(0%)	2(1.79%)	4(2.42%)	3(6.98%)	4.867	0.130
HGB abnormal ^e^ (n, %))	14(3.59%)	5(7.14%)	2(1.79%)	7(4.24%)	0(0%)	4.644	0.157
PLT abnormal ^e^ (n, %)	14(3.59%)	2(2.86%)	4(3.57%)	6(3.64%)	2(4.65%)	0.504	0.935
Baseline anti-HBs(mIU/mL)	117.17(18.60, 429.14)	3.82(1.04, 6.33)	38.65(18.78, 72.56)	337.69(209.44, 525.77)	1000(1000.00, 1000.00)	348.027	< 0.001
End-of-follow-up anti-HBs (mIU/mL)	113.46(9.42, 485.85)	17.67(2.93, 175.37)	61.99(4.49, 296.42)	188.91(28.78, 578.34)	531.11(80.97, 1000.00)	41.535	< 0.001
Anti-HBs decline(n,%)	131(33.59%)	0(0%)	39(34.82%)	61(36.97%)	31(72.09%)	390.00	< 0.001
Liver cirrhosis ^e^ (n, %)	3(0.77%)	0(0%)	1(0.89%)	1(0.61%)	1(2.33%)	2.271	0.513

Note: FIB-4: Fibrosis-4 index, ALT: Alanine Aminotransferase, AST: Aspartate Aminotransferase, TBIL: Total Bilirubin, ALB: Albumin, HGB: Hemoglobin, PLT: Platelet count. b: baseline; e, end-of-follow-up; *P* < 0.05 indicates a statistically significant difference. Groups were defined as follows: Negative group (HBsAb < 10 mIU/mL), low-level group (HBsAb ≥ 10 mIU/mL and < 100 mIU/mL), moderate-level group (HBsAb ≥ 100 mIU/mL and < 1000 mIU/mL), and high-level group (HBsAb ≥ 1000 mIU/mL).Baseline anti-HBs: anti-HBs levels at baseline; End-of-follow-up anti-HBs: anti-HBs levels at the end of follow-up; Anti-HBs decline: Percentage of patients with anti-HBs decline

### Changes in HBsAb during follow-up and the occurrence of adverse events

Based on changes in HBsAb levels during the follow-up period, the study population was divided into three groups: a decline group (131 cases), a stable group (153 cases), and an increase group (106 cases). The incidence of liver-related adverse events during follow-up in the HBsAb decline, stable, and increase groups was 3.05% (4/131), 0.00% (0/154), and 0.00% (0/105), respectively, showing a statistically significant difference (*χ²* = 5.691, *P* = 0.018). Among these four patients, there were two males and two females, and none had cirrhosis at baseline. Detailed information for these patients is presented in Table 3. Correlation analysis between HBsAb level changes and liver-related adverse event incidence revealed a weak but statistically significant negative correlation (rs = -0.125, *P* = 0.014).

**Table 3:** Characteristics of four patients with adverse events

Patient No.	Age (years)	sex	Follow-up duration(months)	anti-HBs ^b^(mIU/ml)	FIB-4 ^b^	Cirrhosis ^b^	anti-HBs ^e^	FIB-4 ^e^	Cirrhosis ^e^
1	44	male	69	21.99	2.65	No	6.07	2.49	No
2	49	male	65	118.03	1.6	No	5.53	3.05	No
3	35	female	111	1000	1.13	No	3.22	0.74	No
4	44	female	70	1000	2.76	No	319.49	3.91	Yes

Note: FIB-4: Fibrosis-4 index; b: baseline; e: end-of-follow-up

**Fig. 1:**
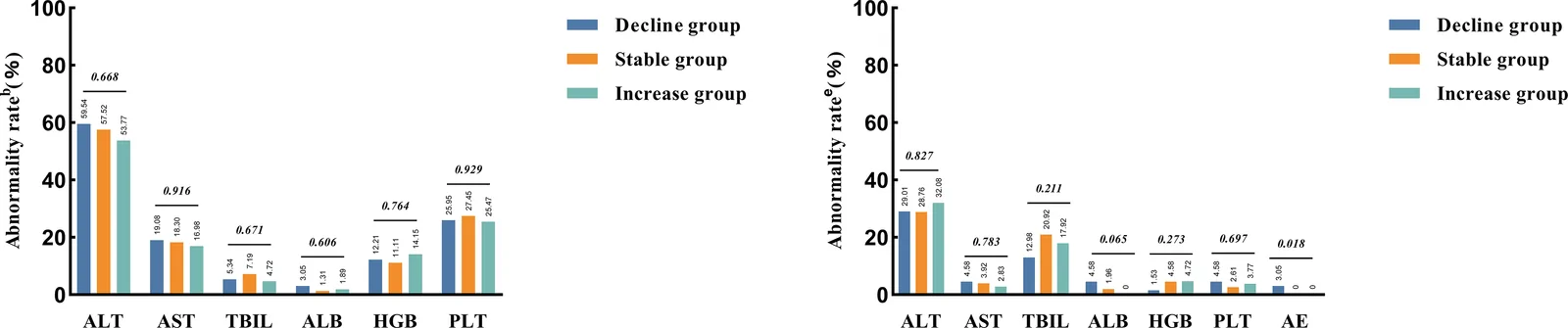
Patient data of the HBsAb decline, stable, and rise groups. Note: Patients were categorized into three groups based on changes in HBsAb levels after discontinuation: decline group, stable group, and rise group. Left panel: Comparison of abnormal rates of clinical indicators among the three groups at treatment discontinuation; Right panel: Changes in abnormal rates of clinical indicators across the three groups by the end of follow-up

At the end of follow-up, a total of 3 patients developed liver cirrhosis, with no significant differences observed among the HBsAb decline, stable, and increase groups [1.53% (2/131) vs. 0.00% (0/154) vs. 0.94% (1/105); *χ²* = 2.244, *P* = 0.370]. Additionally, baseline and endpoint liver function indicators (ALT, AST, TBIL, ALB), HGB levels, and PLT counts showed no significant differences among the three groups. Detailed patient characteristics are presented in Fig. 1.

### Abnormal transaminase levels during follow-up and the development of liver cirrhosis

Neither baseline HBsAb levels nor changes in HBsAb levels during follow-up had a significant impact on the development of liver cirrhosis. By the end of follow-up, only 3 patients had progressed to cirrhosis. Therefore, we further investigated the association between transaminase levels and cirrhosis development.

The incidence of endpoint cirrhosis showed no significant difference between patients with normal and abnormal baseline ALT levels [1.98% (2/167) vs. 0.45% (1/223), *Fisher*, *P* = 0.579] or between those with normal and abnormal baseline AST levels [0.63% (2/319) vs. 1.41% (1/71), *Fisher*, *P* = 0.454]. However, a significant difference was observed in endpoint cirrhosis incidence between patients with normal and abnormal endpoint ALT levels [0.00% (0/274) vs. 2.59% (3/116), *Fisher*, *P* = 0.026], though no such difference existed for endpoint AST levels [0.80% (3/375) vs. 0% (0/15), *Fisher*, *P* > 0.999]. Analysis of the correlation between endpoint cirrhosis incidence and endpoint ALT abnormality rates revealed a weak positive association (rs = 0.135, *P* = 0.026).

## Discussion

Globally, over 325 million people are chronically infected with HBV. Despite the availability of effective vaccines, nearly 1 million individuals die annually from HBV-related complications [8]. Chronic HBV infection significantly increases the risk of developing advanced liver diseases, and HCC is closely associated with HBV infection. It is estimated that approximately 830,000 people worldwide die from liver cancer annually, with 906,000 new cases reported each year [9]. In the Asia-Pacific region, primary liver cancer has the highest incidence rate, ranking as the second leading cause of cancer-related death and the fifth most prevalent cancer type [9].

In patients with chronic HBV infection, long-term antiviral therapy with nucleos(t)ide analogues (NAs) can effectively suppress HBV DNA replication, reverse liver fibrosis and cirrhosis, and reduce the incidence of liver-related adverse events such as HCC and cirrhosis complications. However, the serological clearance of HBsAg remains rare [10, 11]. HBsAg clearance reduces the risk of hepatic decompensation and HCC. Peg-IFNα treatment has been shown to enhance HBsAg seroconversion rates by modulating immune responses, including HBV-specific B lymphocytes and cytotoxic T lymphocytes (CTLs) [12].

CD8-positive T lymphocytes (CD8 + T cells) are critical for controlling HBV infection in the liver by eliminating infected hepatocytes [13]. Single-cell sequencing of peripheral blood has revealed that HBV polymerase 455 (HBV pol455)-specific CD8 + T cells with cytotoxic features—characterized by high expression of granulysin (GNLY), granzyme B (GZMB), perforin 1 (PRF1), and natural killer cell granule protein 7 (NKG7)—are associated with intrinsic HBV control [14]. During chronic infection, virus-specific CD8 + T cells often become dysfunctional and fail to clear infected hepatocytes [15–17]. In patients achieving HBsAg clearance, single-cell sequencing of liver samples demonstrates a significant reduction in exhausted CD8 + T cells expressing programmed death-1 (PD1+), T-cell immunoglobulin and mucin-domain containing-3 (TIM3+), and lymphocyte-activation gene-3 (LAG3+) [18]. These findings suggest that although CD8 + T cells may become functionally exhausted during chronic infection, their activity can be restored upon HBsAg clearance, highlighting their pivotal role in antiviral immunity.

**Fig. 2:**
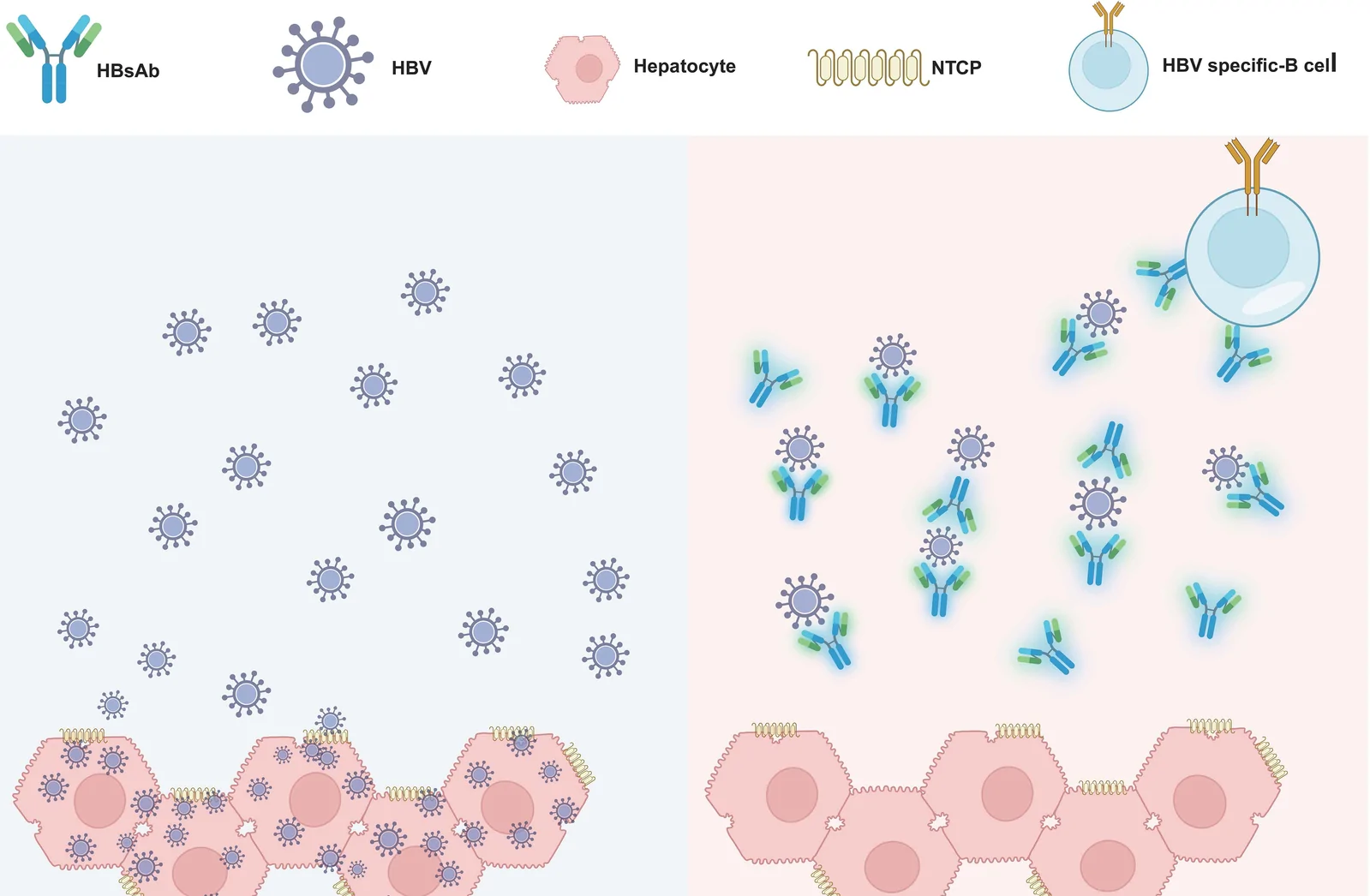
The inhibitory effect of HBsAb on HBV infection. Note: HBsAb: Hepatitis B surface antibody, HBV: Hepatitis B virus, NTCP: Sodium taurocholate co-transporting polypeptide. The left panel shows HBV infecting hepatocytes via the NTCP receptor in the absence of HBsAb; the right panel illustrates that HBsAb inhibits infection by binding to viral surface antigens, thereby blocking their interaction with the NTCP receptor

B lymphocytes play a key role in initiating antiviral immune responses following HBV exposure. Early-activated B cells exhibit upregulated expression of major histocompatibility complex class II (MHC-II), C-X-C motif chemokine receptor 5 (CXCR5), and PD-1, which are closely associated with B cell activation and immune regulation. Peg-IFNα therapy induces an increase in B cell levels, particularly plasmablasts (CD19 + CD38+), which inversely correlate with hepatitis B antigen levels [19]. HBV recovery typically involves two seroconversion events: HBsAg loss accompanied by HBsAb emergence, and HBeAg loss with HBeAb seroconversion. B cells are believed to play a central role in this process [19]. Activated B cells rapidly produce specific antibodies, providing robust immune protection [20, 21]. HBsAb facilitates the clearance of HBV-infected cells through antibody-dependent cell-mediated cytotoxicity (ADCC) and antibody-dependent cellular phagocytosis (ADCP) [22, 23]. Thus, HBsAb positivity typically indicates HBV recovery and HBsAg clearance. However, the relationship between HBsAb and post-treatment liver-related adverse events following HBsAg seroconversion remains unclear. The inhibitory effect of HBsAb on HBV infection is illustrated in Fig. 2.

In this study, the age at HBsAg seroclearance and treatment discontinuation differed significantly among groups with varying HBsAb levels, indicating that higher HBsAb levels were associated with younger ages at treatment discontinuation. HBsAb is secreted by HBV-specific B cells, and its level is closely related to the functional activity of B-cell immunity. During HBV infection, the generation and function of antigen-specific memory B cells are critical for pathogen clearance and long-term immune control [24]. However, B-cell production capacity gradually declines with age, resulting in weakened immune function [24]. This age-related immune decline may explain the negative correlation between HBsAb levels and discontinuation age (rs = -0.248, *P* < 0.001).

In this study, significant differences were observed among groups with different HBsAb levels in terms of HBsAg seroclearance and age at treatment discontinuation, indicating that higher HBsAb levels were associated with younger ages at treatment cessation. HBsAb is secreted by HBV-specific B cells, and its level is closely linked to B-cell immune function. During HBV infection, the generation and function of antigen-specific memory B cells are critical for viral clearance and long-term immune control [24]. However, B-cell production capacity gradually declines with age, leading to impaired immune function [24], which may explain the negative correlation between HBsAb levels and age at treatment discontinuation (rs = -0.248, *P* < 0.001).

Moreover, although bivariate analysis showed a significant association between HBsAb decline and liver-related adverse events, this association did not remain independent of age in regression models. This suggests that dynamic changes in HBsAb may not represent a direct pathogenic factor but rather reflect age-related immune senescence and susceptibility to liver injury. Nevertheless, the decline in HBsAb levels as a biological signal retains important clinical value and may aid in identifying patient subgroups at higher risk due to age-related immune decline, thereby informing individualized management strategies.

HBsAb levels are closely linked to immune activity. Active immune attacks on residual viruses or infected hepatocytes may cause hepatocellular damage, elevating AST and ALT levels [25, 26]. Studies indicate that during Peg-IFNα therapy for CHB, elevated transaminase levels are significantly associated with higher HBsAg clearance rates [27, 28]. Our study found significant differences in abnormal ALT and AST rates among HBsAb-level groups, with higher HBsAb levels associated with increased ALT and AST abnormalities (*rs* = 0.124, *P* = 0.014; *rs* = 0.123, *P* = 0.015). ALT and AST are key markers for liver function assessment, and ALT is generally considered more directly linked to hepatic activity [29]. The observed association between high HBsAb levels and elevated AST in this study may suggest stronger immune activation in patients receiving Peg-IFNα therapy.

In this study, although the occurrence of liver-related adverse events was not associated with baseline HBsAb levels, a significant negative correlation was observed with dynamic changes in HBsAb levels during follow-up (*rs* = -0.125, *P* = 0.014). Notably, a decline in HBsAb level category corresponded to an increased incidence of hepatic adverse events. Among the cohort, four HBsAg-serocleared patients who discontinued treatment experienced liver-related adverse events during follow-up, all occurring in the HBsAb-declining subgroup.

HBsAb-specific B cells have been identified as valuable predictive biomarkers for HBsAg seroconversion in treatment-naïve chronic HBV-infected patients [30]. For those who achieved HBsAg clearance, previous studies demonstrated that HBsAb levels influence the durability of Peg-IFNα-induced HBsAg loss [7, 31, 32]. However, regarding hepatic adverse events, existing research indicates that serological conversion status does not affect the risk of such events [33], consistent with our findings that post-HBsAg clearance, HBsAb levels lack direct correlation with adverse event occurrence. Although baseline antibody levels may partially predict post-HBsAg-clearance relapse, they likely fail to comprehensively reflect dynamic immune system changes or complex virus-host interactions. In contrast, longitudinal HBsAb level alterations directly mirror immune response activity. Our data revealed a significant negative correlation between declining HBsAb levels and liver adverse events, with all four adverse event cases clustered in the HBsAb-declining subgroup. These findings suggest that waning HBsAb levels may weaken immune protection, potentially increasing the risk of viral reactivation or hepatocellular injury.

ALT, a sensitive marker of hepatocellular damage, reflects persistent hepatic inflammation and cellular injury [29]. At the follow-up endpoint, three cirrhosis cases were identified, all occurring in patients with abnormal ALT levels. A positive correlation was observed between the incidence of cirrhosis and the rate of endpoint ALT abnormalities, with cirrhosis prevalence increasing alongside ALT abnormality (*rs* = 0.135, *P* = 0.026). Persistent ALT abnormalities indicate ongoing chronic liver injury, potentially driven by repeated hepatocyte necrosis, hyperactivation of hepatic stellate cells, and aberrant extracellular matrix deposition [34]. This chronic inflammatory microenvironment promotes fibrogenesis, ultimately leading to cirrhosis [35]. These findings highlight the importance of closely monitoring ALT abnormalities in HBsAg-serocleared patients to implement timely interventions that may decelerate cirrhotic progression and improve long-term outcomes.

In this study, 29.74% of patients exhibited abnormal ALT levels at the end of follow-up. These elevations may be attributable to factors such as intense physical activity, recent infections (e.g., cold or fever), medication use (e.g., antipyretics or antibiotics), or insufficient sleep. Although NAFLD was excluded, some patients may have had mild hepatic steatosis or were overweight, which is relatively common in the general population and did not meet the exclusion criteria. Following HBsAg seroclearance, a very small proportion of patients may still experience minimal viral activity or immune fluctuations. Despite the occurrence of mild ALT elevations during follow-up, the risk of severe liver-related events in this population remained low, further supporting the robust long-term protective effect of Peg-IFN–induced HBsAg clearance.

This study has several limitations, including a relatively small sample size and low incidence of hepatic adverse events, potentially affecting statistical power. The study excluded patients with cirrhosis, limiting the generalizability of the findings to chronic HBV populations with advanced liver disease. In addition, other potential confounding factors, including the duration of Peg-IFNα therapy, host genetic background, viral genotype, family history of cirrhosis or hepatocellular carcinoma, and chronic HBV infection duration, were not systematically assessed. Furthermore, non-invasive biomarkers rather than liver biopsy (the diagnostic gold standard) were used for cirrhosis evaluation, introducing potential diagnostic inaccuracies. Future multicenter, large-scale, gold standard-based prospective studies are warranted.

In HBsAg-serocleared, non-cirrhotic patients after treatment cessation, longitudinal HBsAb decline, rather than baseline HBsAb levels, may indicate increased risk of hepatic adverse events. This emphasizes the clinical importance of dynamic HBsAb monitoring to optimize long-term prognosis assessment and management strategies.

## Conclusion

Declining HBsAb levels after HBsAg clearance may be associated with an increased risk of hepatic adverse events.

## Data Availability

Data is provided within the manuscript information files.

## Abbreviations

HBsAb: Hepatitis B surface antibody
HBsAg: Hepatitis B surface antigen
Peg-IFNα: Pegylated interferon-alpha
HBV: Hepatitis B virus
HCC: Hepatocellular carcinoma
HBeAg: Hepatitis B e antigen
HIS: Hospital Information System
LIS: Laboratory Information System
ALT: Alanine Aminotransferase
AST: Aspartate Aminotransferase
TBIL: Total Bilirubin
ALB: Albumin
HGB: Hemoglobin
PLT: Platelet Count
NAs: Nucleos(t)ide analogues
CTLs: Cytotoxic T lymphocytes
CD8 + T cells: CD8-positive T lymphocytes
HBV pol455: HBV polymerase 455
GNLY: Granulysin
GZMB: Granzyme B
PRF1: Perforin 1
NKG7: Natural killer cell granule protein 7
PD1+: Programmed death-1
TIM3+: T-cell immunoglobulin and mucin-domain containing-3
LAG3+: Lymphocyte-activation gene-3
MHC-II: Major histocompatibility complex class II
CXCR5: C-X-C motif chemokine receptor 5
ADCC: Antibody-dependent cell-mediated cytotoxicity
ADCP: Antibody-dependent cellular phagocytosis
NTCP: Sodium Taurocholate Cotransporting Polypeptide
